# Enzyme-Regulated In Situ Formation of Copper Hexacyanoferrate Nanoparticles with Oxidase-Mimetic Behaviour for Colorimetric Detection of Ascorbate Oxidase

**DOI:** 10.3390/bios13030344

**Published:** 2023-03-04

**Authors:** Hao Zhang, Dan-Ni Yang, Yan Li, Feng-Qing Yang

**Affiliations:** 1 Chongqing Key Laboratory of High Active Traditional Chinese Drug Delivery System, Chongqing Medical and Pharmaceutical College, Chongqing 401331, China; 2 School of Chemistry and Chemical Engineering, Chongqing University, Chongqing 401331, China

**Keywords:** copper hexacyanoferrate nanoparticles, oxidase-like activity, ascorbate oxidase, ascorbic acid, colorimetric detection

## Abstract

In this study, a copper hexacyanoferrate nanoparticle with excellent oxidase-mimetic behaviour has been synthesized through a simple precipitation method. The synthesized copper hexacyanoferrate nanoparticle has intrinsic oxidase-like activity, which can catalyze the chromogenic reaction of 2,2′-azinobis-(3-ethylbenzthiazoline-6-sulphonate) through an O_2_^•−^ reactive oxygen-species-participated process. On the other hand, K_3_[Fe(CN)_6_] can be reduced by ascorbic acid (AA) to produce K_4_[Fe(CN)_6_], thereby inhibiting the formation of the copper hexacyanoferrate nanoparticles. Furthermore, ascorbate oxidase (AAO) can catalyze the oxidation of AA to produce dehydroascorbic acid, which cannot reduce K_3_[Fe(CN)_6_]. Thus, a system for an AAO-regulated in situ formation of copper hexacyanoferrate nanoparticles was constructed by coupling a prepared copper hexacyanoferrate nanozyme with AA for the detection of AAO activity. This colorimetric sensing assay shows high sensitivity and selectivity for the detection of AAO activity (the limit of detection is 0.52 U/L) with a linear range of 1.1–35.7 U/L. Finally, the developed method was applied to detect the activity of AAO in normal human serum with a satisfactory sample spiked recovery (87.4–108.8%). In short, this study provides a good strategy for the construction of nanozyme-based multi-enzyme cascade-signal amplification assay.

## 1. Introduction

Ascorbic acid (AA, namely, vitamin C), which is an important nutrient common in humans and animals, plays a vital role in the biochemical metabolism of organisms, through processes such as antioxidation, free radical scavenging, and immune enhancement [1,2,3]. The daily intake level of AA is about 2000 mg according to the Food and Nutrition Committee of the Institute of Medicine of the National Academy of Sciences [4]. However, elevated intake of AA can cause some negative effects, such as urinary stones, taste degradation, and diarrhea [5,6]. On the other hand, insufficient intake of AA is commonly correlated with the incidence of a number of diseases, such as skin diseases and cancer [7]. Ascorbate oxidase (AAO), a versatile copper-containing enzyme, can catalyze the oxidation of AA to dehydroascorbic acid (DHA) to balance the levels of AA in the human body [8]. AAO plays an important role in delaying aging and metabolism by acting as a terminal oxidative enzyme in conjunction with other redox reactions in an organism. In addition, AAO may also be involved in the dynamic homeostasis system of plant oxygen management [9]. Therefore, sensitive methods are highly meaningful to trace and analyze AAO activity.

To date, colorimetry and fluorometry [10,11,12,13] have been reported for the detection of AAO activity. Among them, fluorometry for AAO activity detection has been reported more frequently due to its high sensitivity. Nevertheless, the colorimetry methods associated with the AAO activity detection have rarely been reported. Liu et al. [12] developed a fluorometric and colorimetric detection of AAO activity method through AA-induced growth and aggregation of DNA-templated gold/silver nanoclusters. The limits of detection (LODs) for fluorometric and colorimetric detection of AAO activity are 4.8 U/L and 6.8 U/L, respectively. Wang et al. [13] combined the advantages of ratiometric fluorometry and colorimetry to develop a dual-model strategy for fluorometric and colorimetric detection of AAO activity using carbon dots and *o*-phenylenediamine. The LODs for fluorometric and colorimetric detection of AAO activity are 0.017 U/L and 0.012 U/L, respectively. However, these reported methods usually require expensive fluorescent materials, tedious synthesis processes, and specialized operators.

Nanomaterials with enzyme-like activities (nanozymes) have attracted attention continually since Gao et al. [14] first reported that Fe_3_O_4_ nanoparticles have intrinsic peroxidase-like characteristics. Nanozymes with high catalytic activity are comparable to natural enzymes and have the benefits of high stability, controllable catalytic activity, and flexible functionality over natural enzymes [15,16,17]. Thus far, several nanozymes with peroxidase-like activity have been reported [18,19,20], but they require the addition of H_2_O_2_ as an electron acceptor to oxidize the substrate. It is worth noting that H_2_O_2_ is not only easily decomposed and loses its oxidation ability, but its strong oxidation ability may also destroy the analytes in the constructed sensing system. Alternatively, oxidase-like nanozymes can directly catalyze the oxidation of substrates without H_2_O_2_. For example, Zhan et al. [21] developed a colorimetric method for the detection of dimethoate residue by improving the oxidase-like catalytic activity of cube-shape Ag_2_O, which can directly oxidize the chromogenic substrate of 3,3′,5,5′-tetramethylbenzidine (TMB) to oxidized TMB. Cao et al. [22] developed a ratiometric fluorescence sensor for the detection of glutathione using the oxidase-like activity of a MnO_2_ nanosheet. However, some disadvantages of these nanozymes are nonnegligible, such as the tedious design and synthesis processes. The use of heavy and precious metal materials will pollute the environment and waste resources.

**Figure 1:**
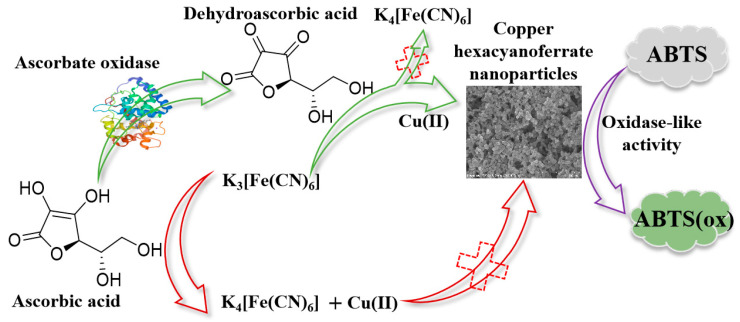
Schematic illustration of enzyme-regulated in situ formation of copper hexacyanoferrate nanoparticles with oxidase-mimetic behaviour for colorimetric detection of ascorbate oxidase.

In this study, a system for an AAO-regulated in situ formation of copper hexacyanoferrate nanoparticles was constructed to detect AAO activity. Considering the development of cost-efficient enzyme mimics with an environmentally friendly and simple synthesis process, the choice was made to synthesize copper hexacyanoferrate nanoparticles. The principle is shown in Figure 1: (1) Unlike the traditional tedious process of nanozyme design and synthesis, copper hexacyanoferrate nanoparticles were synthesized by simply mixing a solution of CuCl_2_·2H_2_O and K_3_[Fe(CN)_6_]; (2) K_3_[Fe(CN)_6_] can be reduced by AA to produce K_4_[Fe(CN)_6_], which can inhibit the formation of the copper hexacyanoferrate nanoparticles; (3) AAO can catalyze the oxidation of AA to generate DHA, decreasing the reduction of K_3_[Fe(CN)_6_] to K_4_[Fe(CN)_6_]. When CuCl_2_·2H_2_O is added, the yield of copper hexacyanoferrate nanoparticles will be increased because of the coordination reaction between Cu(II) and K_3_[Fe(CN)_6_], leading to an increase in the absorption value of oxidized 2,2′-azinobis-(3-ethylbenzthiazoline-6-sulphonate) (ABTS). The oxidase-mimetic behaviour and the catalytic mechanism of copper hexacyanoferrate nanoparticles were investigated by using ABTS as a substrate. A series of experimental parameters, including the enzymatic reaction time between AAO and AA, AA concentration, K_3_[Fe(CN)_6_] concentration, and the enzymatic reaction time between copper hexacyanoferrate nanoparticles and ABTS, were systematically studied. Finally, the developed colorimetric detection method was used to determine the AAO activity in normal human serum.

## 2. Materials and Methods

### 2.1. Chemicals and Materials

Ascorbate oxidase, superoxide dismutase (SOD), L-glutamate sodium, trypsin, and hyaluronidase were obtained from Shanghai Yuanye Bio-Technology Co., Ltd. (Shanghai, China). ABTS, isopropyl alcohol (IPA), K_4_[Fe(CN)_6_], and urea were purchased from Shanghai Macklin Biochemical Co., Ltd. (Shanghai, China). Copper(II) chloride dihydrate (CuCl_2_·2H_2_O) was obtained from Shanghai Titan Scientific Co., Ltd. (Shanghai, China). L-Serine was purchased from Tianjin Guangfu Fine Chemical Research Institute (General Partnership) (Tianjin, China). L(+)-Ascorbic acid, hydrochloric acid (HCl), D-glucose monohydrate, sodium chloride (NaCl), and potassium chloride (KCl) were purchased from Chengdu Chron Chemicals Co., Ltd. (Chengdu, China). Normal human serum was purchased from Beijing Solarbio Science and Technology Co., Ltd. (Beijing, China). Sodium dihydrogen phosphate (NaH_2_PO_4_), K_3_[Fe(CN)_6_], and bovine albumin were obtained from Shanghai Adamas Reagent Co., Ltd. (Shanghai, China).

### 2.2. Instrumentation

An FE 28 pH meter (Mettler-Toledo Instruments, Shanghai, China) was used to measure the pH of the solution. A UC-2H ultrasonic cleaner (Shanghai Titan Scientific Co., Ltd., Shanghai, China) was used to prepare the solution. UV-Vis absorption spectra were recorded with a UV-2600 spectrophotometer (Shimadzu Instruments (Suzhou) Co., Ltd., Suzhou, China). A DHG-9035A drying oven (Shanghai Yiheng Technology Instrument Co., Ltd., Shanghai, China) was used to control the temperature. The crystal structure of the copper hexacyanoferrate nanoparticles was studied by X’Pert Powder X-ray diffraction (XRD) (Empyrean, PANalytical, the Netherlands). Transmission electron microscopy (TEM) images were collected by a JEM-2100F (JEOL, Tokyo, Japan). Scanning electronic microscopy (SEM) images were collected with an SU8100 (Hitachi, Tokyo, Japan).

### 2.3. Preparation of Sample Solutions

K_3_[Fe(CN)_6_] (2.0 mM) and CuCl_2_·2H_2_O (4.0 mM) were prepared by dissolving them in deionized water. The HCl solution (1:50, *v*/*v*) was prepared by diluting concentrated HCl with deionized water. The 0.5 mM of ABTS was prepared in deionized water. AA (0.2 mM) was prepared by dissolving it in phosphate buffer (10.0 mM, pH = 6.0). Different concentrations of AAO were prepared by dissolving AAO in phosphate buffer (10.0 mM, pH 6.0). The interfering substrates of Na^+^, K^+^, Ag^+^, Cl^−^, glucose, L-serine, L-glutamic acid, urea, bovine albumin, trypsin, and hyaluronidase were prepared in deionized water. In addition, 0.2 mg/mL of AA and 2.0 mg/mL of SOD were prepared in deionized water.

### 2.4. Oxidase-Mimetic Behaviour of Copper Hexacyanoferrate Nanoparticles

The oxidase-mimetic behaviour of copper hexacyanoferrate nanoparticles was investigated using ABTS as the substrate. Experiments were performed in the presence of 0.5 mM of K_3_[Fe(CN)_6_], 2.0 μM of CuCl_2_·2H_2_O, and 5.0 mM of ABTS, with a total reaction volume of 225.0 μL. After being incubated at room temperature for 3.0 min, the UV-Vis absorbance spectra of the reaction mixture solution were recorded in the range of 380–500 nm.

### 2.5. Catalytic Mechanism of Copper Hexacyanoferrate Nanoparticles

The catalytic mechanism of the oxidase-mimetic behaviour of copper hexacyanoferrate nanoparticles was investigated. To explore which kind of reactive oxygen species (ROS) was involved in the catalytic process of copper hexacyanoferrate nanoparticles toward ABTS, different radical scavengers, including AA (scavenges •OH and O_2_^•−^), SOD (scavenges O_2_^•−^), and IPA (scavenges •OH), were introduced. A total volume of 260.0 μL of reaction mixture solution containing 100.0 μL of Cu^2+^ solution (1.0 mM), 100.0 μL of K_3_[Fe(CN)_6_] solution (2.0 mM), 10.0 μL of ABTS solution (1.0 mM), and 50.0 μL of varied amounts of radical scavengers were incubated at room temperature for 3.0 min, respectively. Absorbance values at 412 nm were measured.

### 2.6. Kinetic Analysis

The kinetic experiments were performed using 1.0 mM of K_3_[Fe(CN)_6_] and 2.0 mM of CuCl_2_·2H_2_O with different concentrations of ABTS, and absorbance at 412 nm was recorded. The kinetic parameter of the Michaelis–Menten constant (*K*_m_) was obtained according to the Lineweaver-Burk double reciprocal plot: 1/*v* = *K*_m_/(*v*_max_[S]) + 1/*v*_max_, where *v* and *v*_max_ represent the initial reaction velocity and the maximum reaction velocity of the enzymatic reaction, respectively, and [S] is the ABTS concentration.

### 2.7. Determination of AAO Activity Using the Colorimetric Sensing Assay

First, 10.0 μL of deionized water, 10.0 μL of AAO solution, and 50.0 μL of AA solution (0.2 mM) were mixed and reacted at 37 °C for 30.0 min. Then, 10.0 μL of HCl solution, 10.0 μL of K_3_[Fe(CN)_6_] solution (2.0 mM), and 10.0 μL of CuCl_2_·2H_2_O solution (4.0 mM) were added to the above reaction mixture and incubated for 1.0 min at room temperature. Finally, 10.0 μL of ABTS solution (0.5 mM) was added to the above reaction mixture and reacted for 3.0 min at room temperature. The absorbance of the solution at 412 nm was collected using a UV-2600 spectrophotometer. The change in the absorbance value Δ*A* (Δ*A* = *A* − *A*_0_, where *A* and *A*_0_ are the absorbance values of mixture solutions in the presence and absence of AAO, respectively) was calculated, and a calibration curve between AAO activity and Δ*A* was plotted.

### 2.8. Selectivity and Interference Study

The selectivity of the colorimetric method for AAO activity detection was investigated, and the following interfering substrates that may exist in the serum sample were analyzed under the optimal conditions: Na^+^, K^+^, Ag^+^, Cl^−^, glucose, L-serine, L-glutamic acid, urea, bovine albumin, trypsin, and hyaluronidase. In the interference study, these interfering substances were, respectively, mixed with AAO and then analyzed by the UV-Vis spectrophotometer. The initial concentrations of AAO activity and the interfering substances are 0.1 U/mL and 0.5 mg/mL, respectively.

## 3. Results and Discussion

### 3.1. Characterization of the Copper Hexacyanoferrate Nanoparticles

**Figure 2:**
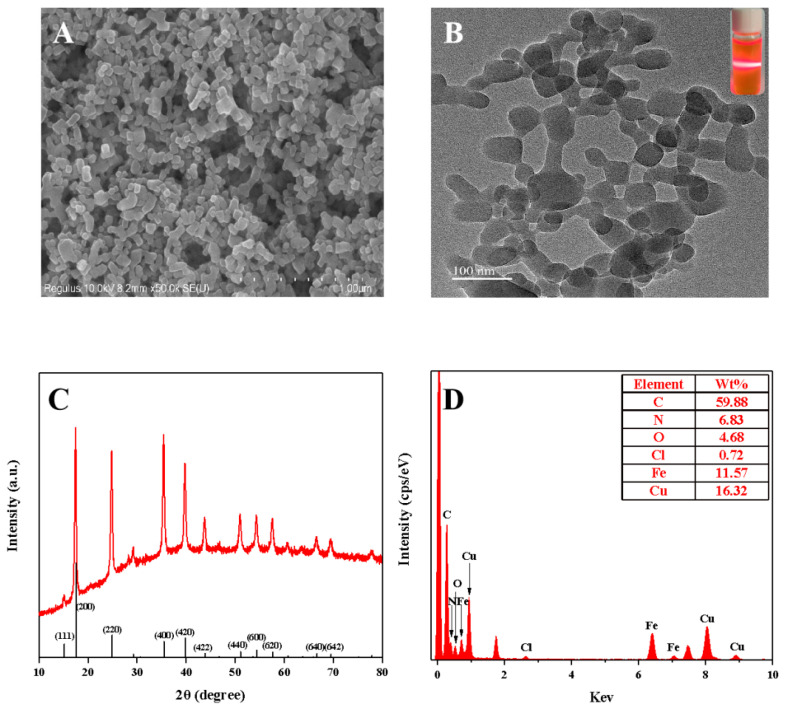
(**A**) SEM and TEM; (**B**) images of the copper hexacyanoferrate nanoparticles (inset: the image of the copper hexacyanoferrate nanoparticles under the irradiation of a red laser); (**C**) XRD plot of the copper hexacyanoferrate nanoparticles; (**D**) EDS plot of the copper hexacyanoferrate nanoparticles.

The copper hexacyanoferrate nanoparticles were synthesized through the coordination reaction between Cu(II) (4.0 mM) and K_3_[Fe(CN)_6_] (2.0 mM) (Figure 1). Figure 2A displays the morphology of the copper hexacyanoferrate nanoparticles investigated through the SEM. Spherical nanoparticles can be observed, indicating the successful formation of copper hexacyanoferrate nanoparticles through the coordination reaction between Cu(II) and K_3_[Fe(CN)_6_]. The synthesized copper hexacyanoate nanoparticles were further characterized using TEM. As shown in Figure 2B, the morphology of nanoparticles is spherical with an average diameter of about 100 nm. When the generated orange-yellow colloid solution was irradiated with a red laser, a clear and straight laser beam passing through the colloid sample can be observed due to the Tyndall effect (insert in Figure 2B), indicating that the nanoparticles are well-dispersed in the aqueous solution. Powder X-ray diffraction (XRD) for nanoparticles was conducted to determine the phase and crystalline pattern. As shown in Figure 2C, eleven main characteristic diffraction peaks can be observed at XRD patterns, which correspond to (111), (200), (220), (400), (420), (422), (440), (600), (620), (640), and (642) lattice planes of Cu_3_[Fe(CN)_6_]_2_, respectively. These main diffraction peaks are well-consistent with the standard JCPDS card No. 70–2702. These results revealed the successful synthesis of copper hexacyanoferrate nanoparticles. Moreover, the surface characteristics of the copper hexacyanoferrate nanoparticles were further characterized by EDX analysis (Figure 2D), and the results indicate the presence of C, N, Fe, and Cu elements on the surface of the copper hexacyanoferrate nanoparticles.

### 3.2. Oxidase-Mimetic Behaviour of Copper Hexacyanoferrate Nanoparticles

**Figure 3:**
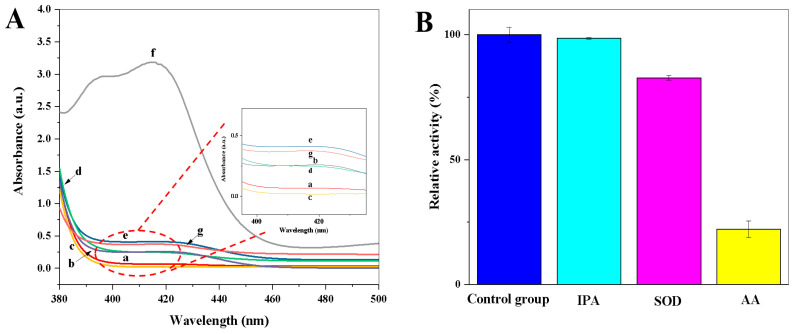
(**A**) UV–Vis absorption spectra of (a) CuCl_2_·2H_2_O solution, (b) K_3_[Fe(CN)_6_] solution, (c) ABTS solution, (d) CuCl_2_·2H_2_O + ABTS mixture solution, (e) K_3_[Fe(CN)_6_] + ABTS mixture solution, (f) CuCl_2_·2H_2_O + K_3_[Fe(CN)_6_] + ABTS mixture solution, (g) CuCl_2_·2H_2_O + K_4_[Fe(CN)_6_] + ABTS mixture solution; (**B**) The relative activity (%) of copper hexacyanoferrate nanoparticles in the presence of different radical scavengers of IPA (scavenges •OH), AA (scavenges •OH and O_2_^•−^), and SOD (scavenges O_2_^•−^) (*n* = 3). The relative activity (%) = the absorbance value in the presence of radical scavengers/the absorbance value in the absence of radical scavengers × 100.

The oxidase-mimetic behaviour of copper hexacyanoferrate nanoparticles was investigated using ABTS as the chromogenic substrate. As shown in Figure 3A, there is no obvious color observed for the CuCl_2_·2H_2_O solution (Figure 3A, a curve); the K_3_[Fe(CN)_6_] solution (Figure 3A, b curve); the ABTS solution (Figure 3A, c curve); and the mixture solutions of CuCl_2_·2H_2_O and ABTS (Figure 3A, d curve) or K_3_[Fe(CN)_6_] and ABTS (Figure 3A, e curve). However, a characteristic absorption peak of oxidized ABTS at 412 nm was observed in the coexistence of CuCl_2_·2H_2_O, K_3_[Fe(CN)_6_], and ABTS (Figure 3A, f curve), indicating that copper hexacyanoferrate nanoparticles have good oxidase-like activity. In addition, the oxidase-mimetic behaviour of the CuCl_2_·2H_2_O, K_4_[Fe(CN)_6_], and ABTS mixture solution was investigated. The CuCl_2_·2H_2_O, K_4_[Fe(CN)_6_], and ABTS mixture solution cannot cause the oxidation of ABTS (Figure 3A, g curve). Thus, the synthesized copper hexacyanoferrate nanoparticles through the coordination reaction between Cu(II) and K_3_[Fe(CN)_6_] in situ can be used as an oxidase mimic to catalyze the oxidation of ABTS.

### 3.3. Catalytic Mechanism of Copper Hexacyanoferrate Nanoparticles

To explore the role of ROS in the catalytic process of copper hexacyanoferrate nanoparticles toward ABTS, different radical scavengers of IPA (scavenges •OH), AA (scavenges •OH and O_2_^•−^), and SOD (scavenges O_2_^•−^) were introduced [23]. As shown in Figure 3B, the change in relative activity (%) is almost negligible after the introduction of IPA, while the relative activity (%) is significantly decreased after the introduction of SOD and AA, respectively. These results indicate that O_2_^•–^ is the main ROS active species involved in the copper hexacyanoferrate nanoparticles-ABTS system.

### 3.4. Kinetic Analysis

The oxidase-mimetic behaviour of copper hexacyanoferrate nanoparticles was investigated by detecting the steady-state kinetic parameter using ABTS as the chromogenic substrate. As shown in Figure S1 (in Supplementary Material), the obtained Lineweaver–Burk double reciprocal plot shows a satisfactory linear correlation (Y = 7.0954X + 0.3182, R^2^ = 0.9841). The *K*_m_ value of copper hexacyanoferrate nanoparticles to the substrate (ABTS) is 22.30 μM. In general, a smaller *K*_m_ value represents a better affinity of the enzyme for the substrate. Compared with previously reported oxidase-like nanozymes (Table S1), the *K*_m_ value of the copper hexacyanoferrate nanoparticles with ABTS as the chromogenic substrate is much lower than other oxidase-like nanozymes, indicating a much higher affinity between the nanoparticles and ABTS.

### 3.5. Feasibility of the Colorimetric Detection of AAO Activity

The feasibility of the colorimetric detection of AAO activity was investigated by several control experiments. As shown in Figure S2, an obvious color (insert in Figure S2) and a characteristic absorption peak of oxidized ABTS at 412 nm was observed in the coexistence of AAO, AA, CuCl_2_·2H_2_O, K_3_[Fe(CN)_6_], and ABTS (Figure S2, a curve), as well as in the coexistence of AAO, CuCl_2_·2H_2_O, K_3_[Fe(CN)_6_], and ABTS (Figure S2, b curve), indicating that AAO can oxidize the AA to produce DHA that cannot reduce the K_3_[Fe(CN)_6_]. In addition, there were no obvious colors observed in the other mixture solutions (Figure S2, c–f curve). Thus, the synthesized copper hexacyanoferrate nanoparticles with oxidase-like activity can be used for the colorimetric detection of AAO activity.

### 3.6. Optimization of the Colorimetric Detection Method

To obtain a good performance of the assay, several experimental parameters were optimized, including the enzymatic reaction time between AAO and AA (5.0–40.0 min), AA concentration (0.07–0.70 mM), K_3_[Fe(CN)_6_] concentration (0.11–0.77 mM), and the enzymatic reaction time between copper hexacyanoferrate nanoparticles and ABTS (0–7.0 min). According to the information provided by the manufacturer, AAO has good activity at pH = 6.0. In addition, the normal human body temperature of 37 °C was selected for the optimization of the colorimetric reaction.

**Figure 4:**
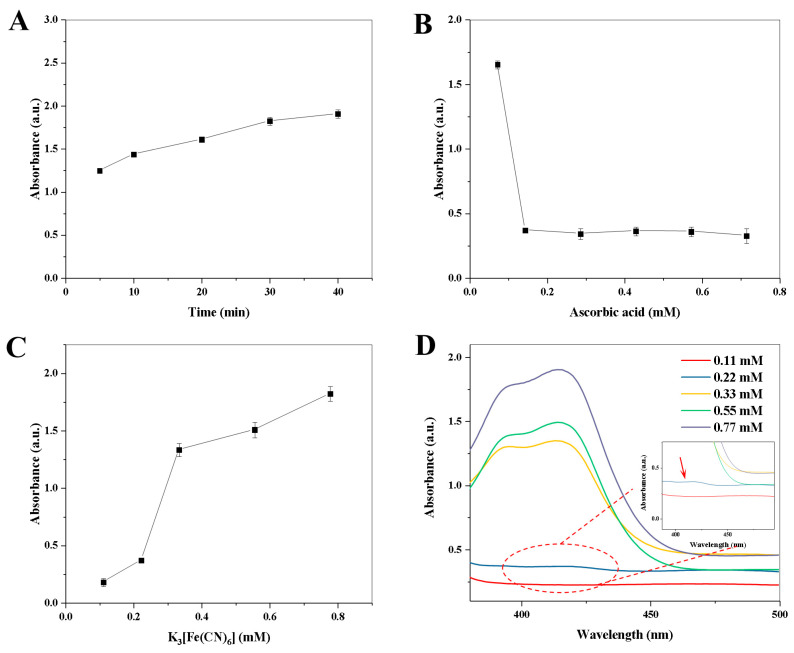
Effects of enzymatic reaction time (**A**) and ascorbic acid concentration (**B**) on the absorbance value at 412 nm.; Effect of K_3_[Fe(CN)_6_] concentration on the absorbance value at 412 nm (**C**), and the corresponding UV–Vis absorption spectra (**D**). Error bars represent standard deviations (*n =* 3).

The effect of enzymatic reaction time between AAO and AA (5.0–40.0 min) on the absorbance value was investigated. As shown in Figure 4A, the absorbance value at 412 nm is increased with the increase in enzymatic reaction time from 5.0 to 30.0 min. However, the absorbance values at 412 nm are almost constant when the enzymatic reaction time exceeded 30.0 min. Therefore, the enzymatic reaction time of 30.0 min was chosen for the next study. Then, the effect of AA concentration (0.07–0.70 mM) on the absorbance value was explored. As shown in Figure 4B, there is a maximum decrement of the absorbance value when the final concentration of the AA solution reaches 0.14 mM, while the absorbance value is almost constant when the AA concentration exceeds 0.14 mM. Therefore, the optimal final concentration of AA is 0.14 mM. Moreover, the variation in absorbance value with the concentration of K_3_[Fe(CN)_6_] (0.1–0.70 mM) and the corresponding spectrogram are shown in Figure 4C,D, respectively. The absorbance value is increased with the increase in the concentration of K_3_[Fe(CN)_6_] from 0.11 to 0.77 mM, and there is a maximum increment of the absorbance value between 0.22 mM and 0.33 mM. To obtain a good sensitivity for AAO detection (Figure 4D), 0.22 mM of K_3_[Fe(CN)_6_] was chosen. Finally, the reaction time between copper hexacyanoferrate nanoparticles and ABTS (0–7.0 min) was investigated. As shown in Figure S3, when the reaction time exceeds 1.0 min, the absorbance value is almost constant. Therefore, 1.0 min of reaction time between copper hexacyanoferrate nanoparticles and ABTS was selected in the following experiments.

### 3.7. Feasibility of the Colorimetric Detection of AAO Activity

**Figure 5:**
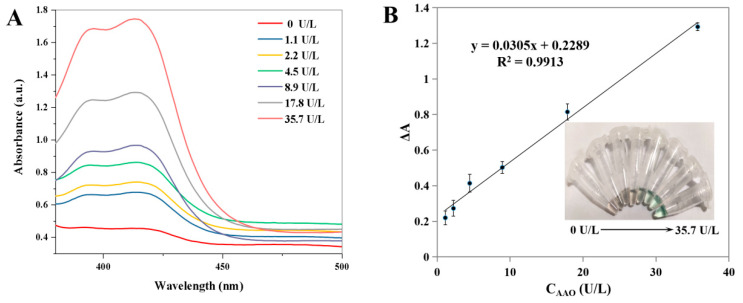
UV–Vis absorption spectra of ABTS system after the addition of different activities of AAO (**A**). The corresponding calibration plots for AAO (**B**) (Insets: the corresponding images). Error bars represent standard deviations (*n =* 3).

**Figure 6:**
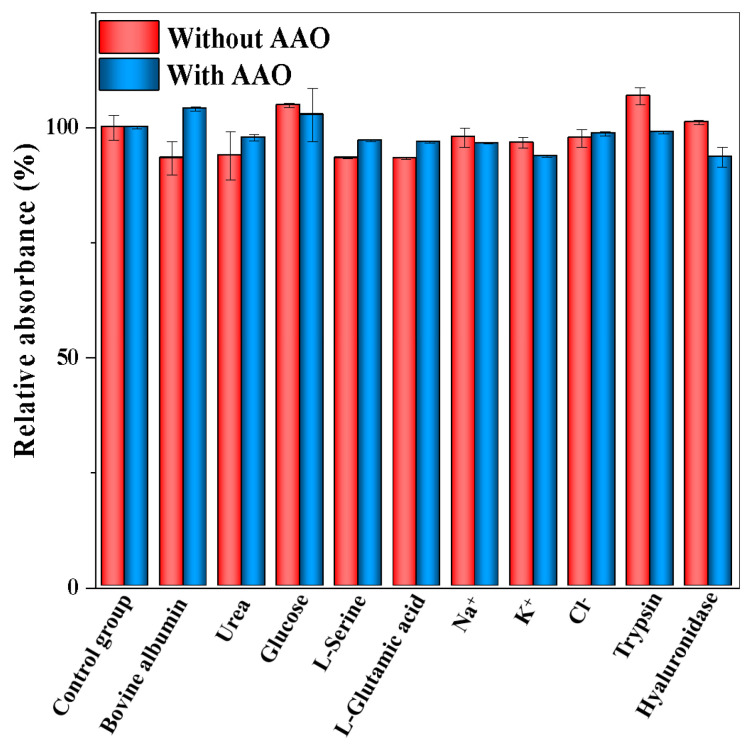
Selectivity and interference study of the colorimetric assay for AAO activity detection. Error bars represent standard deviations (*n =* 3).

As shown in Figure 5A, the absorbance value of ABTS at 412 nm is gradually increased with the increase in the activity of AAO ranging from 0 to 35.7 U/L. The corresponding calibration curve of the change in the absorbance value (Δ*A*) versus AAO activity is shown in Figure 5B. Moreover, different colors appeared with the different activities of AAO (inset in Figure 5B). The linear calibration equation is Δ*A* = 0.0305X + 0.2289 (where X is the AAO activity, Δ*A* is the increment of absorbance value, R^2^ = 0.9913), and the linear range for AAO activity is from 1.1 to 35.7 U/L. The LOD is calculated to be 0.52 U/L according to the 3 *σ*/*S* (where *σ* is the standard deviation, *S* is the slope of the calibration curve). Compared with other AAO detection methods (Table 1), the developed method has a satisfactory linear range and a low LOD value. The selectivity and interference study of the developed method was performed, and the following interfering substrates that may exist in serum samples were analyzed under the optimal conditions: Na^+^, K^+^, Ag^+^, Cl^−^, glucose, L-glutamic acid, L-serine, urea, bovine albumin, trypsin, and hyaluronidase. As shown in Figure 6, no obvious changes in the relative absorbance (%) are observed in the presence or absence of AAO. These results indicate that the colorimetric detection for AAO activity has a satisfactory selectivity.

### 3.8. Real Samples Analysis

The practical application of the nanozyme-based colorimetric method was investigated by sample spiked recovery to detect AAO activity in normal human serum. Before the analysis, normal human serum was diluted 500 times using deionized water. The serum samples were spiked with three different activities of AAO (2.2, 8.9, and 35.7 U/L) and analyzed through the nanozyme-based colorimetric method. In brief, 10.0 μL of the human serum sample, 10.0 μL of AAO solution, and 50.0 μL of AA solution (0.2 mM) were mixed and reacted at 37 °C for 30.0 min. Then, 10.0 μL of HCl solution, 10.0 μL of K_3_[Fe(CN)_6_] solution (2.0 mM), and 10.0 μL of CuCl_2_·2H_2_O solution (4.0 mM) were added to the above reaction mixture and incubated for 1.0 min at room temperature. Finally, 10.0 μL of ABTS solution (0.5 mM) was added to the above reaction mixture and reacted for 3.0 min at room temperature. The absorbance of the solution at 412 nm was collected using a UV-2600 spectrophotometer. The recoveries (Table 2) of the AAO in the human serum sample spiked with three different activities of AAO are in the range of 87.4–108.8%. The results show that the developed method has good reliability in the detection of AAO activity in real samples.

## 4. Conclusions

In summary, a colorimetric detection method for AAO activity was established for the first time through an enzyme-regulated in situ formation of copper hexacyanoferrate nanoparticles-based nanozyme through a simple precipitation method. The synthesized copper hexacyanoferrate nanozyme was coupled with AA to construct an AAO-regulated in situ formation of copper hexacyanoferrate nanoparticles system for the detection of AAO activity with an LOD as low as 0.52 U/L. In short, the colorimetric method proposed in this study has the following merits: (1) it avoids the tedious design and synthesis processes of other nanozymes; (2) the detection process is simple and does not require large equipment; (3) it provides a simple bioanalytical method using a bioenzyme–nanozyme cascade reaction, which has great potential in constructing sensitive cascade-reaction assaying methods.

## Figures and Tables

**Table 1:** Comparisons of the reported methods for the detection of AAO activity.

Materials	Detection Methods	Linear Range (U/L)	LOD (U/L)	Ref.
Copper hexacyanoferrate NPs ^a^	Colorimetry	1.1–35.7	0.52	This study
MoS_2_ QDs ^b^	Fluorimetry	2.0–40.0	0.8	[10]
ZIF-8 ^c^ @QDs and CDs ^d^	Fluorimetry	0.05–4.0	0.02	[11]
Prussian blue NPs	Photothermometry	0.25–14	0.21	[24]
CoOOH nanosheets	Fluorimetry	1.0–20.0	0.25	[25]
Manganese(II)-doped zinc/germanium oxide NPs	Fluorimetry/Scanometric analysis	1250−2500/1000–4000	728/850	[26]
DNA-templated gold-silver nanoclusters	Fluorimetry/Colorimetry	10.0−200.0	4.8/6.8	[12]
Papain-protected bimetallic gold/silver nanoclusters	Fluorimetry	5.0−80.0	1.72	[27]
CDs	Fluorimetry/Colorimetry	0.04–5.0/0.04–8.0	0.017/0.012	[13]
Co-Fe Prussian blue analog nanocube	Colorimetry	0.25–5.0	0.16	[28]

^a^ NPs: nanoparticles; ^b^ QDs: quantum dots; ^c^ ZIF-8: zeolitic imidazolate framework-8; ^d^ CDs: carbon dots.

**Table 2:** Detection of AAO activity in normal human serum using the nanozyme-based colorimetric method (*n* = 3).

Sample	Added (U/L)	Found (SD) (U/L)	Recovery (%) ^a^
Human serum	0	0 ^b^	-
	2.2	2.4 (0.7)	108.8
	8.9	7.8 (1.0)	87.4
	35.7	34.6 (0.6)	96.7

**^a^** Recovery (%) = (found concentration-original concentration)/added concentration × 100. **^b^** The detection value is lower than the detection limit of this method.

## Data Availability

Not applicable.

## Supplementary Materials

The following supporting information can be downloaded at: https://www.mdpi.com/article/10.3390/bios13030344/s1, Figure S1: The double-reciprocal plot of copper hexacyanoferrate nanoparticles. Error bars represent standard deviations (*n* = 3); Figure S2: UV–vis absorption spectra of (a) AAO + AA + K_3_[Fe(CN)_6_] + CuCl_2_·2H_2_O + ABTS mixture solution, (b) AAO + K_3_[Fe(CN)_6_] + CuCl_2_·2H_2_O + ABTS mixture solution, (c) AA + K_3_[Fe(CN)_6_] + CuCl_2_·2H_2_O + ABTS mixture solution, (d) AAO + AA + CuCl_2_·2H_2_O + ABTS mixture solution, (e) AAO + AA + K_3_[Fe(CN)_6_] + ABTS mixture solution, (f) AAO + AA + K_3_[Fe(CN)_6_] + CuCl_2_·2H_2_O mixture solution (Insets: the corresponding images); Figure S3: The effect of reaction time between copper hexacyanoferrate nanoparticles and ABTS on the absorbance value at 412 nm. Error bars represent standard deviations (*n* = 3); Table S1: Comparison of apparent Michaelis−Menten Constant (*K*_m_) of copper hexacyanoferrate nanoparticles for the oxidation of ABTS with other reported oxidase-like nanozymes. References [29,30,31,32,33] are cited in the Supplementary Materials.
