# Supplementary material for: Enzyme-Regulated In Situ Formation of Copper Hexacyanoferrate Nanoparticles with Oxidase-Mimetic Behaviour for Colorimetric Detection of Ascorbate Oxidase
Source: Biosensors (Basel). 2023 Mar 4;13(3):344. doi: 10.3390/bios13030344 (PMC10046506; doi:10.3390/bios13030344)
Supplement: Supplementary file 1 [file biosensors-13-00344-s001.zip › biosensors-2242976-supplementary.pdf]

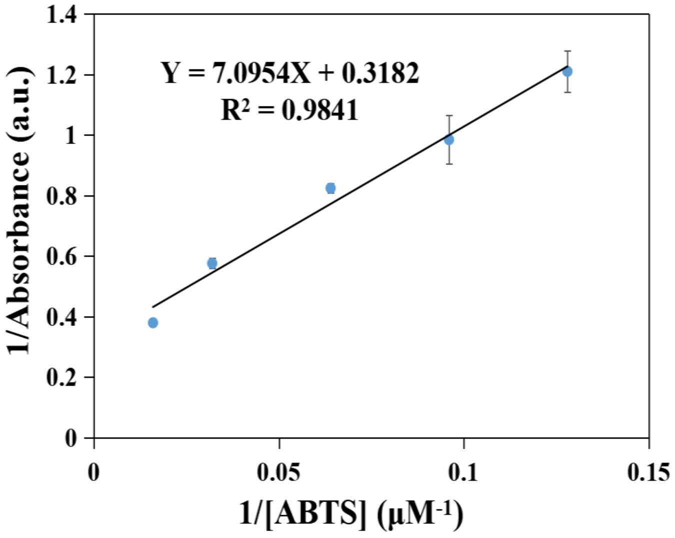

Figure S1. The double-reciprocal plot of copper hexacyanoferrate nanoparticles. Error bars represent standard deviations ($n = 3$).

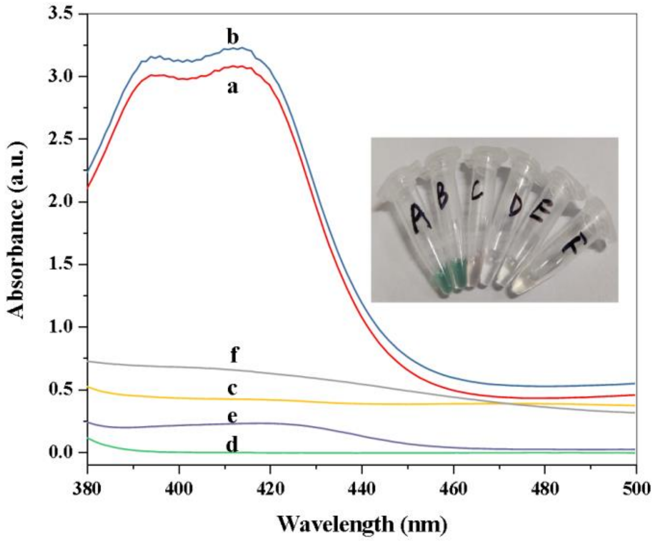

Figure S2. UV–vis absorption spectra of (**a**) AAO+AA+$K_3[Fe(CN)_6]$+$CuCl_2{\cdot}2H_2O$ +ABTS mixture solution, (**b**) AAO+$K_3[Fe(CN)_6]$+$CuCl_2{\cdot}2H_2O$+ABTS mixture solution, (**c**) AA+$K_3[Fe(CN)_6]$+$CuCl_2{\cdot}2H_2O$+ABTS mixture solution, (**d**) AAO+AA+$CuCl_2{\cdot}2H_2O$+ABTS mixture solution, (**e**) AAO+AA+$K_3[Fe(CN)_6]$ +ABTS mixture solution, (**f**) AAO+AA+$K_3[Fe(CN)_6]$+$CuCl_2{\cdot}2H_2O$ mixture solution (Insets: the corresponding images).

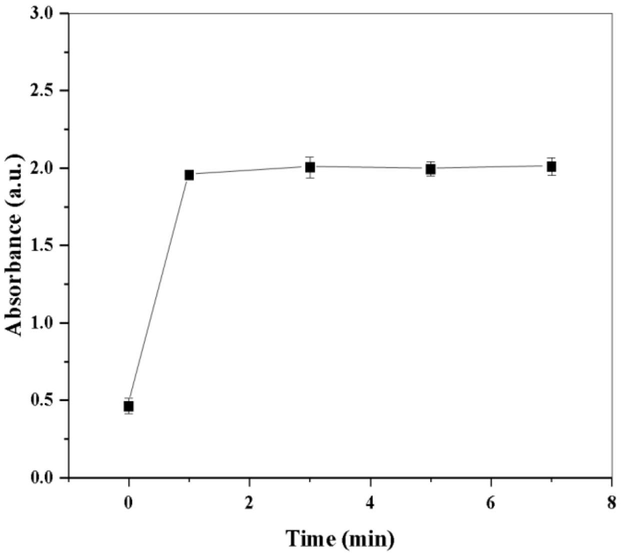

Figure S3. The effect of reaction time between copper hexacyanoferrate nanoparticles and ABTS on the absorbance value at 412 nm. Error bars represent standard deviations (*n* = 3).

Table S1. Comparison of apparent Michaelis–Menten Constant (K_m) of copper hexacyanoferrate nanoparticles for the oxidation of ABTS with other reported oxidase-like nanozymes.

Oxidase-like nanozymes	K_m (μM)	Ref.
Copper hexacyanoferrate NPs [a]	22.30	This study
MoO_3 NPs	1676.9	[29]
UiO-66-NH_2@Ce	720	[30]
AuNCs [b]-GMP/PtNCs-GMP/Au−PtNCs-GMP	95.3/1440/132.1	[31]
Co_3O_4 NPs	37	[32]
Ag NPs	7.2	[33]

[a] NP: nanoparticles; [b] NCs: nanocluster.
